# Roles of trait resilience, flexibility, and volitional self-control in social adaptation: An fMRI study

**DOI:** 10.1192/j.eurpsy.2021.1945

**Published:** 2021-08-13

**Authors:** S. Tei, J. Fujino

**Affiliations:** 1 Department Of Psychiatry, Kyoto University, Kyoto, Japan; 2 Institute Of Applied Brain Sciences, Waseda University, Saitama, Japan; 3 School Of Human And Social Sciences, Tokyo International University, Saitama, Japan; 4 Department Of Psychiatry And Behavioral Sciences, Tokyo Medical and Dental University, Tokyo, Japan

**Keywords:** flexibility, self control, resilience, fMRI

## Abstract

**Introduction:**

Resilience and cognitive flexibility are considered as pre-adaptive traits that help individuals to deal with environmental and social distress. Although they may alleviate emotional impulses and/or support volitional self-control (VSC), these cognitive mechanisms remain insufficiently explored.

**Objectives:**

To better understand resilience and flexibility, we built a mechanistic framework to explain variations in socially adaptive responses under distressing situations (cooperation dilemma) using economic and social decision-making paradigms.

**Methods:**

Twenty-four university students (7 females) were enrolled. We used ego-resiliency (ER) and Machiavellian (Mach) questionnaires to measure resilience and flexibility, and applied third-party punishment (TPP) and ultimatum game (UG), as well as moral dilemma (MD) tasks to derive VSC-associated brain activity using 3T-functional magnetic resonance imaging. Mediation analysis was used to investigate whether these pre-adaptive trait levels explain cooperative decision-making (invested sum in TPP and acceptance rate of unfair offers in UG), together with VSC-associated brain activity during MD. The regions of interest included the orbitofrontal cortex (OFC), dorsolateral prefrontal cortex (DLPFC), and temporoparietal junction (TPJ).

**Results:**

Pre-adaptive traits were a statistically significant mediator for two different models. There was an indirect effect of 1) ER on the relationship between OFC activity strength and TPP scores, and 2) Mach on the relationship between DLPFC/TPJ and UG (p < 0.05).

**Conclusions:**

Although recruitment is still ongoing, our results suggested that trait resilience and flexibility may help other-regarding and goal-directed motivation shifts. They may align self-interests with collective interests and support VSC, thereby adjusting peoples’ behaviors within social contexts and cultivating social intelligence.

**Disclosure:**

No significant relationships.

